# Neurofibromatosis Type 1: A Novel NF1 Mutation Associated with Mitochondrial Complex I Deficiency

**DOI:** 10.1155/2014/423071

**Published:** 2014-03-04

**Authors:** Sara Domingues, Lara Isidoro, Dalila Rocha, Jorge Sales Marques

**Affiliations:** ^1^Pediatrics Department, Centro Hospitalar do Tâmega e Sousa, EPE, Unidade Padre Américo 4564-007 Penafiel, Portugal; ^2^Pediatrics Department, Centro Hospitalar de Vila Nova de Gaia/Espinho, EPE, Unidade II, 4400-129 Vila Nova de Gaia, Portugal

## Abstract

*Background.* Neurofibromatosis type 1 is a multisystemic, progressive disease, with an estimated incidence of 1/3500-2500. Mitochondrial diseases are generally multisystemic and may be present at any age, and the global prevalence is 1/8500. The diagnosis of these disorders is complex because of its clinical and genetic heterogeneity. *Case Report.* We present a rare case of the association of these two different genetic diseases, in which a heterozygous missense mutation in the NF1 gene was identified which had not yet been described (p.M1149 V). Additionally, the patient is suspected of carrying an unspecified mutation causing respiratory chain complex I deficiency. Clinical presentation included hypotonia, global development delay, reduced growth rate, progressive microcephaly, and numerous *café-au-lait* spots. *Discussion.* To the best of our knowledge this is the first report of complex I deficiency in a patient with neurofibromatosis type 1. It is very important to maintain a high index of suspicion for the diagnosis of mitochondrial disorders. In this patient, both the laboratory screening and muscle histology were normal and only the biochemical study of muscle allowed us to confirm the diagnosis.

## 1. Introduction

Neurofibromatosis type 1 (NF1), first described in 1882 by von Recklinghausen [1, 2], is a multisystemic [1, 3], progressive disease [2], with an estimated incidence of 1/3500-2500 [1–4]. In about half of the cases, it is an autosomal dominant inherited disorder, and in the remaining cases, it results from *de novo* mutations [1, 3]. It has high penetrance and variable phenotypic expression between and within families [1]. It results from mutations in the NF1 tumor suppressor gene located on chromosome 17 [2], responsible for encoding neurofibromin [1]. The three main characteristics of this disease are *café-au-lait* spots, multiple neurofibromas, and Lisch nodules (pigmented *hamartomas* of the Iris) [5].

Mitochondrial disorders are a heterogeneous group of diseases characterized by defects of mitochondrial structure and oxidative phosphorylation [6–8]. These disorders are generally multisystemic [6, 8] and may be present at any age [7], and the global prevalence, probably underestimated, is 1/8500 [7]. The organs with highest energy demand, such as, heart, brain, skeletal muscle tissue, and liver, are preferentially involved [6, 8–11]. Treatment is supportive [7, 12] and does not influence the natural course of the disease [8, 13], and the prognosis is often poor [11, 14]. Mitochondrial complex I deficiency is the most common defect of the oxidative phosphorylation system [10]. The diagnosis of mitochondrial disease is complex because of its clinical and genetic heterogeneity [7, 12, 14]. We present a case report on a child with NF1 and deficiency of the mitochondrial complex I, an association not described before.

## 2. Case Report

The patient, a boy, was born after a normal pregnancy of 39 weeks, through an instrumented vaginal delivery. His birth weight was 2970 g. At birth, he had a low Apgar score (3/6/8 at the first, fifth, and tenth minute of life, resp.), hypotonia, grunt, and respiratory depression, requiring noninvasive ventilation. During neonatal intensive care stay, he maintained hypotonia and feeding difficulties. Inborn errors of metabolism (including twenty four diseases of three main groups: aminoacidopathies, organic acidemias, and mitochondrial fatty-acid oxidation disorders) and congenital hypothyroidism were not detected in the analysis of the Guthrie card. The metabolic study (plasma lactate, pyruvate, ammonia, amino acids, carbohydrate deficient transferrin, acylcarnitines, and urinary organic acids profile plus amino acids) and brain magnetic resonance (MRI) were normal. He was discharged from hospital at 28 days of life to continue followup as an outpatient and was referred to a developmental early intervention program.

Family history was notable for the mother, uncles, and grandmother having various *café-au-lait* spots, negative for Leigh syndrome or mitochondrial dysfunction.

Regarding the milestones, he just reached the sitting position after twelve months. Gait and first words were present at twenty-four months. At three years of age, he maintained low weight and short stature for his age, hypotonia, and global development delay. Physical examination revealed progressive microcephaly, more than six *café-au-lait* spots, greater than 0.5 cm in diameter, (these appeared in the first year of life and increased progressively in number and size) and a systolic murmur.

Molecular study confirmed an heterozygous mutation in the NF1 gene (mutation c.3445A>G(p.Met1149Val)-exon 26). Liver, renal, and hemopoietic function were normal along with plasma lactate, pyruvate, amino acids, and urinary organic acids profile. Magnetic resonance spectroscopy and deltoid muscle histology were normal. The analysis of enzymatic activity of mitochondrial respiratory chain complexes I–V in muscle (spectrophotometric assay) revealed partial (29%) deficit of complex I activity relative to citrate synthase (5.7, normal: 8.8–30.8). In the molecular research, we used the technique of *polymerase chain reaction* followed by direct sequencing of genes encoding subunits of complex I. The most common mutations and mitochondrial DNA deletions of large size (3243A>G, 3271T>C, 8344A>G, 8356T>C, and 8993T>C/G) (5, 10, and 11) were sent to research. Then we studied the seven mitochondrial genes (MTND1-MTND6, and MTND4L) and subsequently sequenced the eleven nuclear genes in which mutations have been so far described—NDUF1, NDUFS2, NDUFS3, NDUFS4, NDUFS6, NDUFS7, NDUFS8, NDUFV1, NDUFV2, and NDUFA1 NDUFA8. All the molecular study was negative. Cardiac evaluation detected a low grade pulmonary stenosis; cardiomyopathy and arrhythmia were not observed. Subclinical hypothyroidism (thyroid-stimulating hormone 7.45 *μ*UI/mL, normal: 0.27–4.2; free thyroxine 1.18 ng/dL, normal: 0.93–1.7; antithyroid peroxidase and thyroglobulin antibodies: negative) was also detected. The remaining studies (karyotype including fluorescence in situ hybridization, screening for celiac disease, and abdominal and pelvic ultrasound) were normal. Ophthalmologic and audiologic evaluations were normal.

He started treatment with coenzyme Q_10_ plus levothyroxine and maintained dietary supplementation as well as occupational and speech therapies.

At the age of six, the brain MRI revealed subthalamus lesions of probable hamartomatous nature. At this age, evaluation with the Ruth Griffiths scale revealed global developmental delay involving uniformly motor, verbal, and cognition skills (general developmental quotient: 73%). According to the classification of the Diagnostic and Statistical Manual of Mental Disorders Fifth Edition (DSM-V) and the Conner's Ratting Scales, the patient did not meet criteria for attention-deficit/hyperactivity disorder (ADHD).

**Figure 1:**
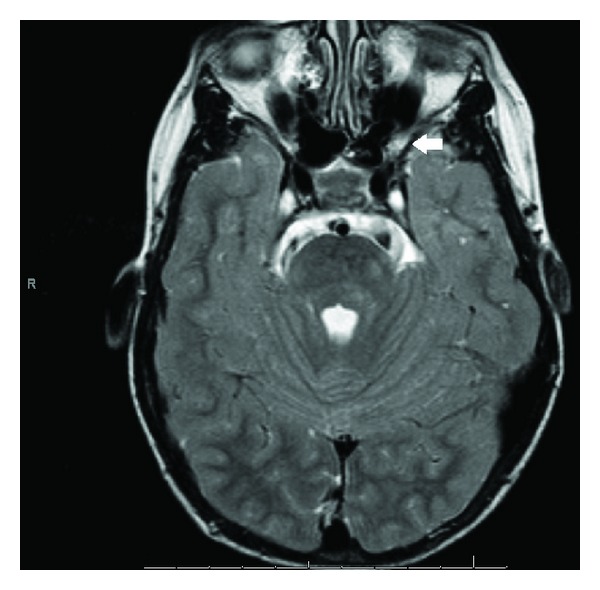
Brain MRI: axial section (T2) showing optic nerve glioma.

Currently, he is eight years old and is clinically well and asymptomatic. Nevertheless, cranial magnetic resonance imaging revealed a left optic nerve glioma in its prechiasmatic segment (Figure 1). After evaluation by a multidisciplinary team, it was decided to pursue a conservative approach with regular clinical and neuroimagiological followups (at 3 to 12 months intervals). There is no tumor progression until now in our patient.

## 3. Discussion

The authors present a child with NF1 in which a heterozygous missense mutation in the NF1 gene was identified that had not yet been described. Since NF1 is a very common disease [2], its association with other diseases is likely to occur coincidentally. Nevertheless, to the best of our knowledge, this is the first report of complex I deficiency in a patient with NF1.

These two diseases share important characteristics: they are both multisystemic and progressive [2, 7]. It is thus important for the physician to be alert to the characteristic signs and symptoms. Patients with NF1 are not expected to present microcephaly [2], and if it occurs, they should be appropriately investigated. It is among these atypical cases that mitochondrial disease is more common [7, 13]. It would thus be prudent to evaluate such patients with unexplained combination of multisystem symptoms and progressive clinical course for possible mitochondrial disease [15]. Consequently, we suggest that screening for mitochondrial disorders is included in the investigation of microcephaly in patients with NF1.

These diseases are frequently associated with neurocognitive deficits, including ADHD, autism spectrum disorders, behavioral abnormalities, and psychosocial issues [4, 14]. We highlight the importance of early (prescholar) and complete evaluation in these patients, to prepare the child, family, and technical education in the prevention of learning difficulties, promoting self-esteem, and social integration [2].

Optic nerve glioma developed in approximately 15% of patients with NF1 [1–3, 16]. It is the more feared complication, and also the one that leads to more doubts regarding treatment and followup [2]. It generally occurs before eight years of life and may present with visual impairment, facial asymmetry, proptosis, strabismus, and endocrine signs or symptoms (elevated growth velocity, precocious puberty) [2, 16]. In patients with NF1, optic pathway gliomas are typically low-grade pilocytic astrocytomas [3, 4] and grow more insidiously [16], leading to a better prognosis than in other patients [1, 3]. In our patient, the tumor was not progressive or clinically significant. Thus, as recommended by different authors [1, 16], a conservative approach, with regular followup was elected. If the disease becomes progressive, the patient should start treatment with chemotherapy [1, 4]. Radiotherapy is contraindicated [1, 4] and surgical treatment is not recommended unless the lesion exhibits rapid growth or the patient's clinical state deteriorates [4].

It is very important to maintain a high index of suspicion for the diagnosis of mitochondrial disease, because most patients do not present easily recognizable disorders [15]. Except for some specific mitochondrial encephalomyopathic syndromes, the clinical features are rarely pathognomonic for the diagnosis of mitochondrial disorders and symptoms can be difficult to assimilate into a unifying diagnosis particularly in the case of pediatric patients [15]. Pediatric mitochondrial disorders can be accompanied by normal muscle morphology, normal plasma lactate, normal mitochondrial enzymes on skeletal muscle, normal mitochondrial DNA mutation screening, and a nonclassical clinical presentation because none of these criteria has absolute sensitivity to detect mitochondrial disease [11]. In the present patient, both the laboratory screening and muscle histology were normal and only the biochemical study of muscle allowed us to confirm the diagnosis. It is well known that mitochondrial respiratory chain enzymatic defects are not specific, because they are also found in other disorders [14]. Therefore it is difficult to ascertain in this case if the enzymatic defect results from a primary or secondary cause [14].

Mitochondrial complex I deficiency is the most common defect of the oxidative phosphorylation system [10]. Most cases result from autosomal recessive inheritance; less frequently the disorder is maternally inherited or sporadic and the genetic defect is in the mtDNA [9]. At present, the diagnosis is often only based on biochemical measurements of the single enzyme activities of the oxidative phosphorylation system as the genetic cause is still unknown in many patients [8, 10]. The investigation of the deficits of complex I is highly complex due to the large number of subunits that comprise it, thus hindering the molecular characterization of these patients [8]. In our center it is intended to extend the molecular study to known genes encoding processing factors (NDUFAF1 and B17.2L) as well as those that encode subunits of complex I, in which no mutations have been described to date, hoping that further investigation will allow us to increase the number of patients with definite diagnosis [8]. Enhanced methods are important to identify new mutations causing complex I deficiency; they may be useful tools in the near future, improving genetic counseling and prenatal diagnosis in at-risk families [8, 10].

Arun et al. [17] demonstrated a novel interaction between neurofibromin and leucine-rich pentatricopeptide repeat motif-containing protein, which subsequently links NF1 and Leigh syndrome French Canadian variant, at the molecular level. They further show that this interaction occurs as part of a ribonucleoprotein complex consistent with RNA granules, which are important epigenetic regulators. Further studies into the etiopathogenesis of NF1 and Leigh syndrome and mitochondrial dysfunction may contribute to our understanding of the molecular mechanisms that contribute to the complex developmental phenotypes associated with these syndromes.

## References

[1] Couto C, Monteiro T, Araújo L, Temudo T (2012). Neurofibromatosis type 1: diagnosis and follow-up in paediatrics. *Acta Pediátrica Portuguesa*.

[2] Martins CL, Monteiro JP, Farias A, Fernandes R, Fonseca MJ (2007). Managing children with neurofibromatosis type 1: what should we look for?. *Acta Médica Portuguesa*.

[3] Pascual-Castroviejo I, Pascual-Pascual SI, Velazquez-Fragua R, Viaño J (2012). Corpus callosum tumor as the presenting symptom of neurofibromatosis type 1 in a patient and literature review. *Revista de Neurologia*.

[4] Williams VC, Lucas J, Babcock MA, Gutmann DH, Bruce B, Maria BL (2009). Neurofibromatosis type 1 revisited. *Pediatrics*.

[5] Chinnery PF, Pagon RA (1993). Mitochondrial disorders overview. *GeneReviews*.

[6] Ferreiro-Barros CC, Tengan CH, Barros MH (2008). Neonatal mitochondrial encephaloneuromyopathy due to a defect of mitochondrial protein synthesis. *Journal of the Neurological Sciences*.

[7] Challa S, Kanikannan MA, Jagarlapudi MMK, Bhoompally VR, Surath M (2004). Diagnosis of mitochondrial diseases: clinical and histological study of sixty patients with ragged red fibers. *Neurology India*.

[8] Ferreira M, Aguiar T, Vilarinho L (2008). Cadeia respiratória mitocondrial aspectos clínicos, bioquímicos, enzimáticos e moleculares associados ao défice do complexo I. *Arquivos de Medicina*.

[9] The United Mitochondrial Disease Foundation Mito profile complex I information. http://www.umdf.org/atf/cf/%7B8d4a231c12fb4a219a8593c7bd0c5a5a%7D/COMPLEX_1_DEFICIENCY.PDF.

[10] Hoefs SJG, van Spronsen FJ, Lenssen EWH (2011). *NDUFA10* mutations cause complex I deficiency in a patient with Leigh disease. *European Journal of Human Genetics*.

[11] Scaglia F, Towbin JA, Craigen WJ (2004). Clinical spectrum, morbidity, and mortality in 113 pediatric patients with mitochondrial disease. *Pediatrics*.

[12] Mattman A, O'Riley M, Waters PJ (2011). Diagnosis and management of patients with mitochondrial disease. *BC Medical Journal*.

[13] Naviaux RK (1997). Overview the spectrum of mitochondrial disease. *Mitochondrial and Metabolic Disorders: A Primary Care Physician's Guide*.

[14] Diogo L, Grazina M, Garcia P (2009). Pediatric mitochondrial respiratory chain disorders in the centro region of Portugal. *Pediatric Neurology*.

[15] Costa RL, Martha AS, Steffen N, Martha VF (2009). Neurofibromatose tipo 1 em criança com manifestação parafaríngea. *X Salão de Iniciação científica—PUCRS*.

[16] Binning MJ, Liu JK, Kestle JRW, Brockmeyer DL, Walker ML (2007). Optic pathway gliomas: a review. *Neurosurgical Focus*.

[17] Arun V, Wiley JC, Kaur H, Kaplan DR, Guha A (2013). A novel neurofibromin (NF1) interaction with the leucine-rich pentatricopeptide repeat motif-containing protein links neurofibromatosis type 1 and the French Canadian variant of Leigh's syndrome in a common molecular complex. *Journal of Neuroscience Research*.

